# A clinical study of thermal monitoring techniques of ultrasound-guided microwave ablation for hepatocellular carcinoma in high-risk locations

**DOI:** 10.1038/srep41246

**Published:** 2017-01-23

**Authors:** Han Zhi-yu, Liang Ping, Yu Xiao-ling, Cheng Zhi-gang, Liu Fang-yi, Yu Jie

**Affiliations:** 1 The Departments of Interventional Ultrasonics, General Hospital of Chinese PLA, Fuxing Road 28. 100853, Beijing, China

## Abstract

To confirm the safety and effectiveness of the minimally invasive thermal monitor technique on percutaneous ultrasound-guided microwave ablation (MWA) for hepatocellular carcinoma (HCC) in high-risk locations, a total of 189 patients with 226 HCC nodules in high-risk locations were treated with MWA. The real-time temperature of the tissue between the lesion margin and the vital structures was monitored by inserting a 21G thermal monitoring needle. The major indexes of technical success, technique effectiveness, local tumour progression and complications were observed during the follow-up period. Technical success was acquired in all patients. Technique effectiveness was achieved with one session in 119 lesions based on contrast-enhanced ultrasound (CEUS) 3–5 days after treatment. An additional 95 lesions achieved technique effectiveness at the second session. Within the follow–up period of 6–58 months (median 38 months), the 1-, 2-, 3-, and 4-year local tumour progression rate was 11.1%, 18.1%, 19.1%, and 19.9%, respectively. There were no major complications in all the patients except for the common side effects. These results indicate that the thermal monitor technique can be applied to prevent major complications in vulnerable structures and allow percutaneous MWA to achieve satisfactory technique effectiveness in the treatment of HCC in high-risk locations.

Hepatocellular carcinoma (HCC) is one of the most prevalent human cancers worldwide, with 626,000 estimated new cases annually and almost as many deaths. Furthermore, 82% of cases (and deaths) occur in developing countries (with 55% in China)^1^. HCC most often arises in a background of cirrhosis, rendering many patients as less than ideal candidates for liver resection because of the limited hepatic reserve. Moreover, the tendency for HCC to develop in the remaining cirrhotic liver makes image-guided thermal ablation a minimally invasive procedure that could readily be reapplied, which is highly desirable for many patients and providers^2^,^3^. Temperature is the parameter governing tissue destruction in thermal ablation, and it induces cellular death via thermal coagulation. The measurement of temperature during thermal therapy provides an accurate estimate of the region of thermal damage that will occur in the tissue. Although thermal ablation has a low complication rate, it can be hazardous when the tumour is in close contact with vulnerable structures^4^. The major complication rates have been shown to reach 4.1 and 4.6% for radiofrequency ablation (RFA) and MWA, respectively,^5^. Biliary tract damage includes bile duct injuries, biliary stricture, bilomas and, most rarely, bilioperitoneum and biliopleural fistula. The incidence of biliary tract damage could be as low as 0.1 and up to 12%^6^. Perforation of the gastrointestinal tract has been reported as a serious complication of thermal ablation, with an overall incidence of 0.1–0.3%^7^. There are several appropriate techniques for protecting vital structures adjacent to tumours from thermal injury, and most of these techniques (such as dextrose solution or saline injection, carbon dioxide dissection, balloon interposition, etc.) are suitable for protecting the gastrointestinal tract^8^,^9^. However, reliable temperature information during clinical hyperthermia and thermal ablation are essential for adequate treatment control, both in the tumour and at potential hot spot locations in the normal tissue^10^. Thermocouples are widely used because of their valuable characteristics including cost-effectiveness, accuracy and wide measurement range. Moreover, their small size and rapid response make thermocouples suitable for localized monitoring of temperature with fast changes^11^. A superfine thermocouple-needle system can monitor the temperature in real-time during an ablation procedure. Placing thermocouples at the intended ablation margin permits exact thermal ablation^12^. In the authors’ institution, thermocouples have been used to monitor and modify the temperature adjacent to the hepatic hilum or gastrointestinal tract^13^,^14^,^15^. To confirm the safety and effectiveness of this procedure, we retrospectively evaluated this minimally invasive thermal monitoring technique on percutaneous ultrasound-guided MWA for HCC in high-risk locations including the gastrointestinal tract, the second or third bile ducts and the gallbladder.

## Results

### Baseline of Patients

The study group included 149 males and 40 females (mean age 58.9 ± 11 years, range 25–86 years). The size of the lesions ranged from 0.9 to 5.9 cm (mean maximum diameter 2.7 ± 1.1 cm). The lesions were adjacent to the gallbladder in 58 lesions (Group GB), the second or third bile ducts in 69 lesions (Group BD), and the gastrointestinal tract in 99 lesions (Group GI). The mean maximum diameter of the lesions was 2.5 ± 1.0 cm, 2.7 ± 1.0 cm, and 2.8 ± 1.2 cm in Group GB, BD and GI, respectively. No significant difference in the maximum diameters was found among Group GB, BD and GI (*P* = 0.18).

### Outcome of Microwave Ablation

The temperature cut off for MWA was set at 55 °C in the gastrointestinal tract and bile ducts and 60 °C in the gallbladder. When the temperature measured by the thermal monitoring needle reached the set temperature, the emission of MW was stopped immediately and was restarted when the temperature dropped to lower than 45 °C. This process continued until the entire lesion was completely covered by the hyperechoic region on the grey-scale US and the total time with the temperature above 54 °C was more than 3 min. During the treatment session, the measured temperature fluctuated between 45 °C and 65 °C.

**Figure 1:**
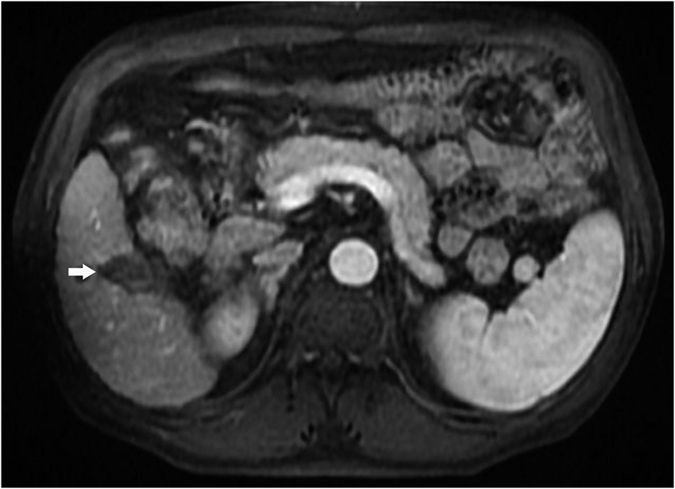
Lesion (white arrow) near the gastrointestinal tract after ablation as shown with enhanced MRI.

**Figure 2:**
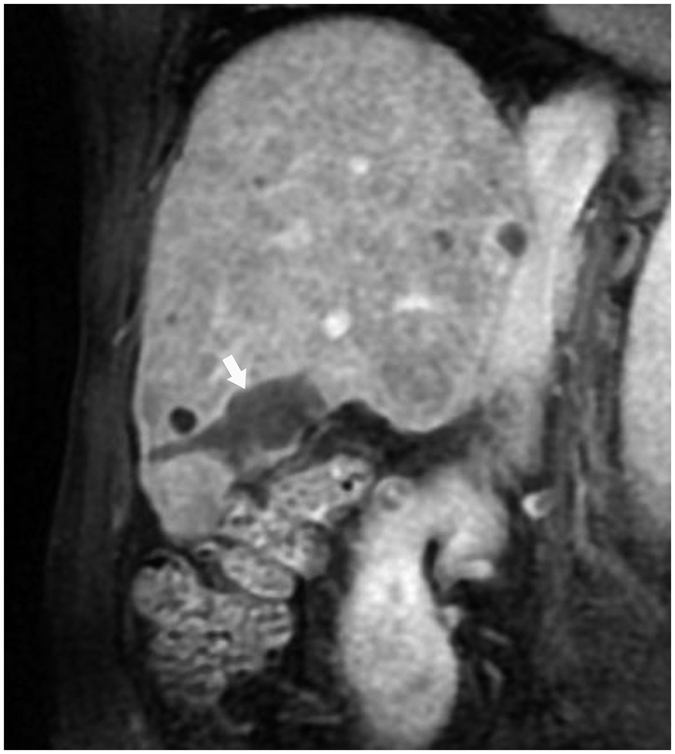
Lesion (white arrow) near the gastrointestinal tract after ablation as shown with enhanced MRI in the coronal view.

**Figure 3:**
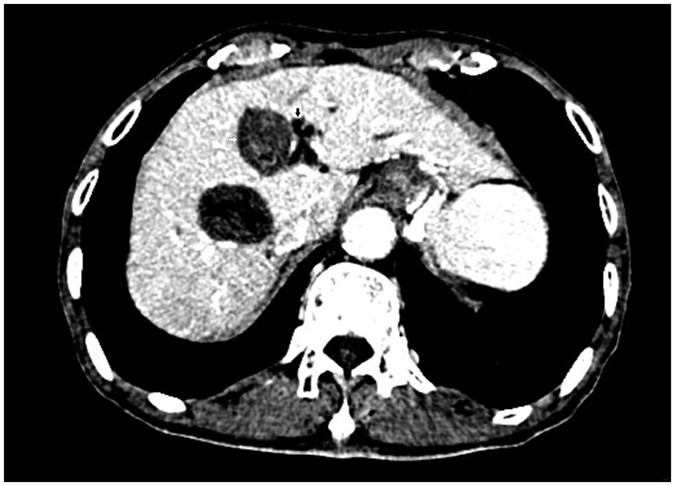
Lesion (white arrow) pushing the left bile duct (black arrow) after ablation as shown with enhanced CT.

**Figure 4:**
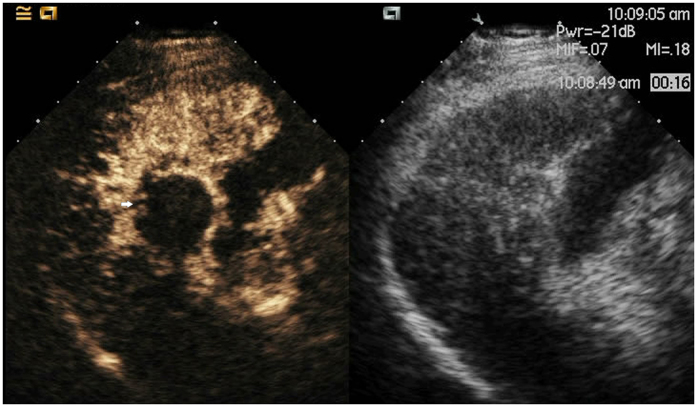
Lesion (white arrow) pushing the gallbladder after ablation as shown with CEUS.

**Figure 5:**
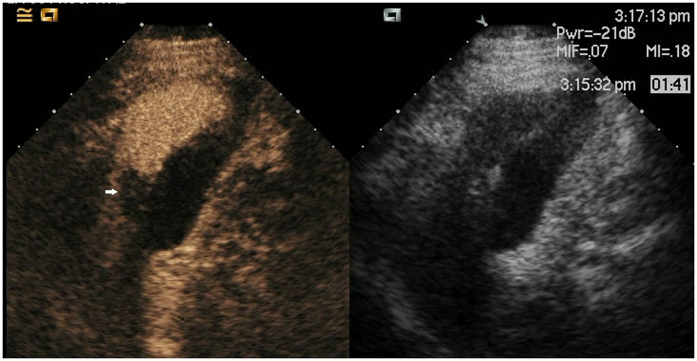
Lesion (white arrow) pushing the gallbladder after ablation approximately 3 years as shown with CEUS.

Technical success was acquired in all 189 patients. Technique effectiveness after one session was achieved in 119 lesions (52.7%, 119/226) based on CEUS 3–5 days after treatment. An additional 95 lesions (42.0%, 95/226) achieved technique effectiveness at the second session. The remaining lesions (5.3%, 12/226) were re-examined within 3 months in which 4 lesions were confirmed to have residual viable tumour tissue within the treatment zone. The rate of complete ablation was 98.2% (222/226) (Figs 1,2,3,4 and 5).

### Local Tumour Progression

Within the follow–up period of 6–58 months (median 38 months), the 1-, 2-, 3-, and 4-year local tumour progression rate was 11.1%, 18.1%, 19.1%, and 19.9%, respectively. The maximum diameter of 45 local tumour progression lesions was 3.5 cm ± 1.1 cm and that of the no local tumour progression lesions was 2.5 cm ± 0.9 cm. There was a significant difference between the two groups of tumours (*P* = 0.00). There was no significant difference in the cumulative incidence of local tumour progression among three groups (*P* = 0.44, log-rank test).

### Complications

There were neither immediate nor periprocedural and delayed major complications in any of the patients. No tumour seeding occurred, particularly at the site of the thermal monitoring needle. The side effects included pain, post-ablation syndrome, and minimal asymptomatic perihepatic fluid or blood collection seen on imaging.

## Discussion

Image-guided (e.g., fluoroscopy, US, CT, and MR imaging) ablative therapy, which encompasses the expertise in imaging technology and various tumour ablation techniques, is one of the most rapidly expanding fields in radiology and medicine. Thermal ablation is a widespread minimally invasive procedure for achieving local control of various malignant tumours, particularly in liver cancers^16^,^17^. Temperature is the parameter governing tissue destruction in thermal ablation, and it induces cellular death via thermal coagulation. Temperatures greater than 60 °C are associated with uniform tissue necrosis^18^,^19^. During energy deposition, the actual tissue temperature is usually difficult to assess. To improve the therapeutic efficiency and safety of the intervention, real-time mapping of temperature and thermal dose appear to offer the best strategy to optimize thermal ablation interventions and provide clinical therapy endpoints^20^. RFA devices can only measure the temperatures in the direction of the RFA electrode axis after treatment. However, the ablated areas usually spread concentrically around the electrode and are normally smaller in the direction perpendicular to the RFA electrode axis than in the direction parallel to the RFA electrode axis^12^.

At present, MR imaging is the only modality with well-validated techniques for real-time temperature monitoring^16^. However, technical difficulties related to motion artefacts and quality control in MR temperature maps, and/or the need for real-time availability of temperature data has limited the use of temperature mapping^20^. Furthermore, because MR systems are highly susceptible to interference from the RF ablation device, appropriate filtering has to be implemented^21^ or ablation and MR data acquisition need to be interleaved^10^,^22^. Moreover, the initial cost of a facility and the routine operation of a magnet remains an impediment to its widespread application in the emerging field of thermal ablation. Therefore, the development of alternative, less costly, clinical imaging systems/techniques to guide heat delivery in real time is presently a priority^23^.

The US imaging systems possess ideal qualities of a guidance technique due to several advantages, such as widespread availability, real-time guidance for electrode placement and accurate and convenient puncture guides^16^. However, a significant drawback of current ultrasound methods is that the zone of necrosis produced during thermal ablation is not easily visualized because of the low intrinsic contrast between the normal and ablated tissues and the artefacts from gas bubble formation^24^. The transient hyperechoic region represents the microbubbles of water vapour that are a result of tissue vaporization during active heating. Although the monitoring of the echogenic response may be most useful in providing a rapid and convenient approximation of the ablation size, it is not a precise marker because both under- and overestimation of the true extent of coagulation have been reported^25^,^26^,^27^,^28^,^29^. In the present study, we could thus estimate with relative accuracy whether a sufficient and safe margin of ablation could be obtained by placing the thermal monitoring needle at the tissue between the lesion margin and the vital structures perpendicular to the MWA antenna axis.

Although CT is also an effective way to monitor thermal ablation, CT scanners do not have real-time capability, which is a disadvantage of precise probe positioning. Moreover, other limitations to the use of CT for thermal ablation include fixed imaging in the axial plane. This can lead to difficulty ablating tumours under the diaphragm or in other areas where oblique imaging planes are desirable. In addition, the use of CT exposes both the patient and physician to ionizing radiation^26^,^27^.

One limitation of the present study was that no control lesions were tested in addition to the high-risk locations. Because the ablation of HCC in high-risk locations does not provide a 0.5- to 1-cm “safety margin” in every direction, the use of control lesions in general locations was not possible. Moreover, artificial ascites was not applied in this study when HCC was treated near the gastrointestinal tract because the local ascites was not always capable of dividing a safety zone.

In conclusion, these results indicate that the technique of minimally invasive thermal monitoring can be applied to prevent major complications in the vulnerable structures and achieve satisfying technique effectiveness for percutaneous MWA in the treatment of HCC in high-risk locations. Further research is needed in randomized studies with a longer follow-up and large sample size.

## Methods

### Patients

A total of 189 patients with 226 HCC nodules were treated with MWA from January 2009 to March 2014 and then followed up continuously until December 2014.

All treatments were performed at our institution with the approval of the Institutional Ethics Committee. Written informed consent was obtained from all patients at the time of enrolment in the study after the nature of the ablation procedure, and possible complications were fully explained.

The diagnostic criteria for HCC was based on the recommendations of the European Association for the Study of the Liver^30^, which are based on ultrasound-guided biopsy, the concordant classical dynamic radiological features of HCC in two radiologic techniques, or one radiologic technique showing typical features of HCC together with an elevated α fetoprotein (AFP) level over 400 ng/mL.

**Figure 6:**
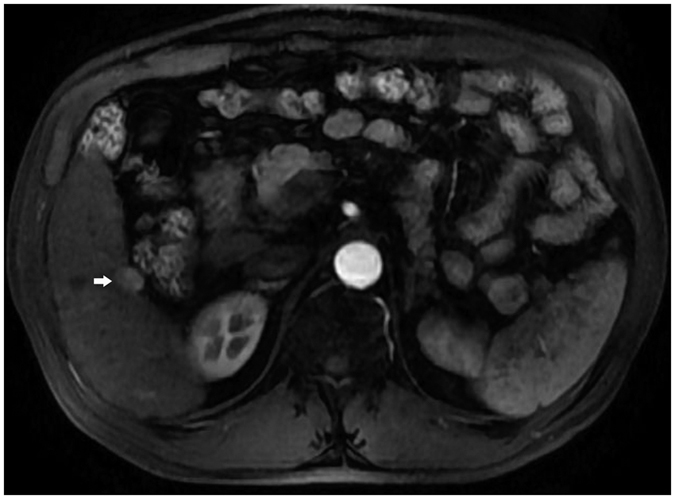
Lesion (white arrow) near the gastrointestinal tract as shown with enhanced MRI.

**Figure 7:**
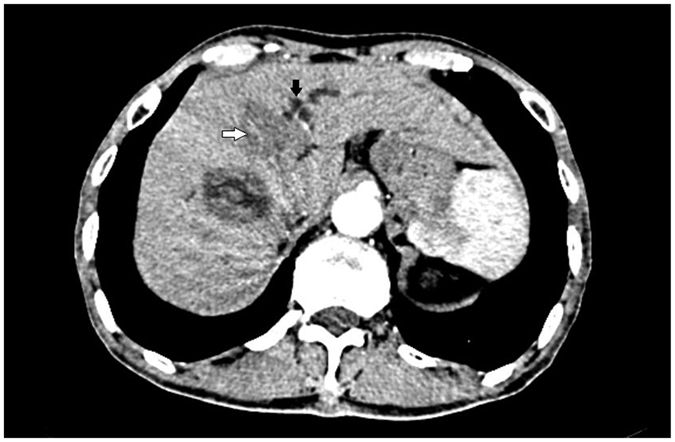
Lesion (white arrow) pushing the left bile duct (black arrow) as shown with enhanced CT.

**Figure 8:**
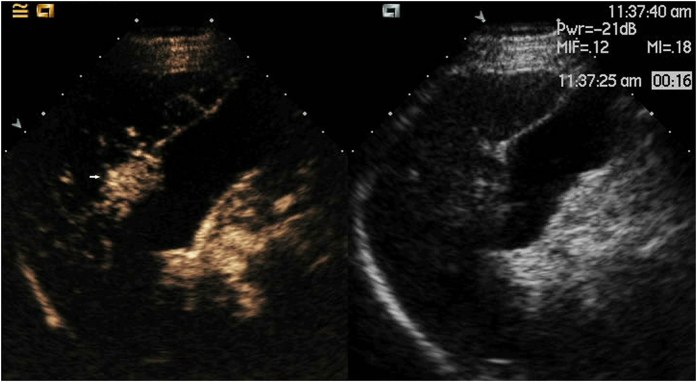
Lesion (white arrow) pushing the gallbladder as shown with CEUS.

The inclusion criteria for tumours in high-risk locations in the present study were defined as a lesion (distance less than 5 mm) near the gastrointestinal tract (Group GI) (Fig. 6), the second or third bile ducts (Group BD) (Fig. 7) or the gallbladder (Group GB) (Fig. 8) based on the CEUS and enhanced MRI or CT before the ablation procedure. The other inclusion criteria were as follows: (1) a single lesion less than 6 cm; (2) no extrahepatic metastasis or vascular tumour invasion; (3) an appropriate percutaneous puncture route as presented on US; and (4) Child-Pugh classification of liver function of A or B, with prothrombin time < 30 s, prothrombin activity > 30%, and platelet count > 40 × 10^9^/L.

### Equipment

The microwave ablation system (KY2000, Kangyou Medical Instruments, Nanjing, China) consisted of two independent MW generators, two flexible coaxial cables and two cooled shaft antennae. The generator was capable of producing 1 to 100 W of power at 2,450 MHz, which could drive up to 1 antenna. The cooled shaft antenna had a 15-gauge shaft coated with polytetrafluoroethylene to prevent adhesion. The distance from the aperture of the MW emission to the needle tip was 11 mm and the emission aperture was 1 mm.

**Figure 9:**
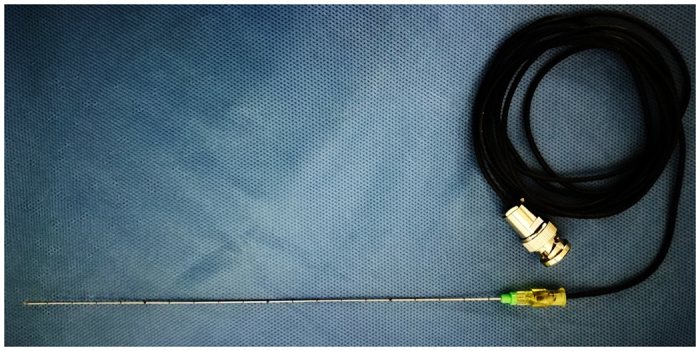
A picture of the thermal monitoring needle with its wire.

A thermal monitoring system attached to the microwave ablation unit was used during treatment. The thermal monitoring needle (Kangyou Medical Instruments, Nanjing, China) was made of a thermocouple embedded in the tip of 21-G percutaneous transhepatic cholangiography (PTC) needle (Fig. 9). The accuracy of the thermocouple was corrected to less than 0.1 °C. The temperature data were recorded using the microwave ablation system.

CEUS, contrast-enhanced CT and/or MR were used to evaluate the ablative response and follow-up study. The US and CEUS were performed using the Sequoia US system (Acuson, Mountain View, CA, USA) with 3.5–5.0 MHz linear multifrequency transducer. The ultrasound contrast agent was Sonovue (Bracco Company, Milan, Italy). All CT studies were conducted with the same multi-detector row CT (Lightspeed 16, GE Medical Systems, Milwaukee, WI, USA) and contrast medium (iopromide, Ultravist 300, Schering, Berlin, Germany). All MR studies were performed with the same 1.5-T unit (Signa Echo-Speed, GE Medical Systems) and contrast medium (Magnevist, Schering; 0.1 mmol/kg body weight).

### Techniques

**Figure 10:**
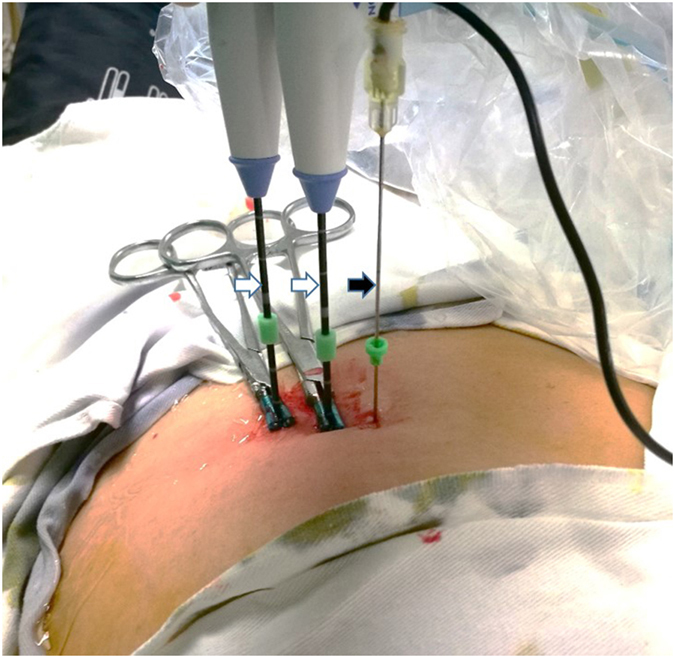
The appearance of the antennae (white arrow) and the thermal monitoring needle (black arrow) after they were placed in the body of the patient.

**Figure 11:**
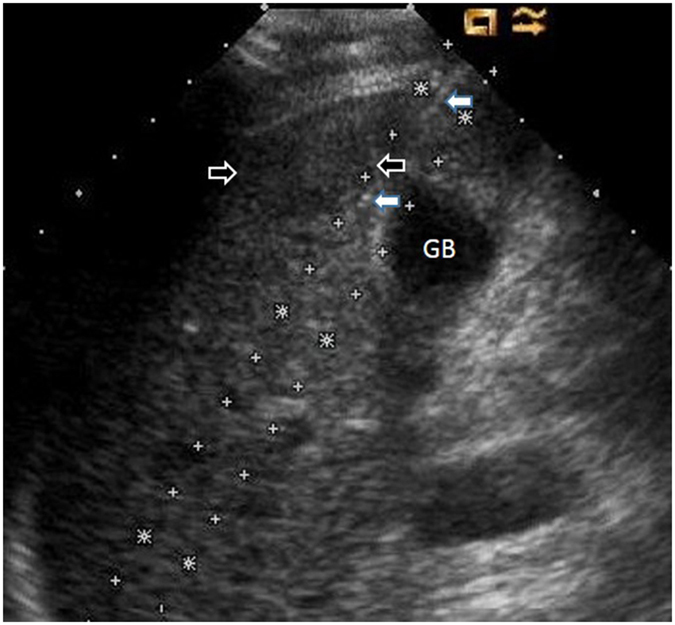
The thermal monitoring needle (white arrow) was inserted into the tissue between the lesion (black arrow) and the gallbladder using US guidance.

All the 189 patients were treated according to the established protocol. The treatment protocol was designed according to the ablation range and the tumour location. After local anaesthesia with 1% lidocaine (Shuanghe Pharmaceuticals, Beijing, China), MW antennae were inserted through a skin incision with US guidance. A general anaesthetic (Propofol, 2.0 mg∕kg, Astra Zeneca, S.P.A. Italy) was applied after proper placement of the antennae, and then MW was emitted. A power output of 45 W to 60 W was used during MWA. One or two thermal monitoring needles were inserted into the tissue between the lesion margin and the high-risk location structures (Figs 10 and 11). The highest temperature near the dangerous structures was set according to experimental and clinical experiences from our previous study^13^,^14^,^15^. To acquire complete ablation for lesions larger than 3 cm or very close to the dangerous structures, a small dose of ethanol was applied as adjuvant therapy. The absolute ethanol was slowly injected into the marginal tissue of the tumour using a 21 G PTC needle during the ablation. When withdrawing the antennae, the needle tracks were routinely cauterized to avoid bleeding and tumour seeding.

### Follow-up

To evaluate the tumour response to MWA (technique effectiveness), CEUS was used to detect any residual viable tumour within 3 days after the treatment. Technique effectiveness was considered to be achieved if the scans revealed the non-enhancing area only covered the tumour border and there was no irregular hyper-enhancing area in the primary tumour. If residual viable tumour was found, another session was performed immediately. If the residual viable tumour was indeterminate after the second session, the patient was re-examined within 3 months. After MWA, CEUS and contrast-enhanced CT or MRI was repeated at 1-month and 3-month intervals within 1 year and then at 6-month intervals. Complete ablation was defined as no residual viable tumour tissue within the treatment zone as confirmed by CEUS, spiral triphasic enhanced MRI or CT performed 3 months after MWA. Local tumour progression was defined as the appearance of viable tumour tissue that was contiguous with the area completely ablated during the follow-up period^30^. The major complications were defined as gallbladder or gastrointestinal perforation, bile duct stricture and peritonitis.

### Statistical Analysis

Data analysis was performed using SPSS for windows (Version 16.0). The patient age and tumour size were expressed as the mean ± standard deviation (SD), and the time of follow-up was expressed as the median. Independent samples t-tests and one-way ANOVAs were used to compare the means between two groups and among the three groups. The cumulative incidence of local tumour progression for the three groups was calculated using the Kaplan-Meier technique, and differences were tested using the log-rank test. *P* values of less than 0.05 indicated statistical significance.

## Additional Information

**How to cite this article**: Zhi-yu, H. *et al*. A clinical study of thermal monitoring techniques of ultrasound-guided microwave ablation for hepatocellular carcinoma in high-risk locations. *Sci. Rep.* **7**, 41246; doi: 10.1038/srep41246 (2017).

**Publisher's note:** Springer Nature remains neutral with regard to jurisdictional claims in published maps and institutional affiliations.
